# Distances and angles in standing long-leg radiographs: comparing conventional radiography, digital radiography, and EOS

**DOI:** 10.1007/s00256-024-04592-9

**Published:** 2024-02-21

**Authors:** Christof Birkenmaier, Louise Levrard, Carolin Melcher, Bernd Wegener, Jens Ricke, Boris M. Holzapfel, Andrea Baur-Melnyk, Dirk Mehrens

**Affiliations:** 1 Department of Orthopaedics and Trauma Surgery, Musculoskeletal University Center Munich (MUM), University Hospital, LMU Munich, Großhadern Campus, Marchioninistr. 15, 81377 Munich, Germany; 2 Artemed Klinikum München Süd, Department for Spine Surgery and Scoliosis Center, Am Isarkanal 30, 81379 Munich, Germany; 3 EOS Imaging, 10 rue Mercoeur, 75011 Paris, France; 4 Schön Klinik Neustadt, Klinik für Wirbelsäulenchirurgie und Skoliosezentrum, Am Kiebitzberg 10, 23730 Neustadt, Germany; 5 Department of Radiology, LMU University Hospital, LMU Munich, Großhadern Campus, Marchioninistr. 15, 81377 Munich, Germany; 6 Radiologie München, Burgstraße 7, 80331 Munich, Germany

**Keywords:** Conventional radiography, Digital radiography, Fan-beam, Cone-beam, Slot-scanner, EOS, Lower extremity, Varus, Valgus, Projectional distortion, Distortion

## Abstract

**Objective:**

Distances and angles measured from long-leg radiographs (LLR) are important for surgical decision-making. However, projectional radiography suffers from distortion, potentially generating differences between measurement and true anatomical dimension. These phenomena are not uniform between conventional radiography (CR) digital radiography (DR) and fan-beam technology (EOS). We aimed to identify differences between these modalities in an experimental setup.

**Materials and methods:**

A hemiskeleton was stabilized using an external fixator in neutral, valgus and varus knee alignment. Ten images were acquired for each alignment and each modality: one CR setup, two different DR systems, and an EOS. A total of 1680 measurements were acquired and analyzed.

**Results:**

We observed great differences for dimensions and angles between the 4 modalities. Femoral head diameter measurements varied in the range of > 5 mm depending on the modality, with EOS being the closest to the true anatomical dimension. With functional leg length, a difference of 8.7% was observed between CR and EOS and with the EOS system being precise in the vertical dimension on physical-technical grounds, this demonstrates significant projectional magnification with CR-LLR. The horizontal distance between the medial malleoli varied by 20 mm between CR and DR, equating to 21% of the mean.

**Conclusions:**

Projectional distortion resulting in variations approaching 21% of the mean indicate, that our confidence on measurements from standing LLR may not be justified. It appears likely that among the tested equipment, EOS-generated images are closest to the true anatomical situation most of the time.

## Introduction

Uniplanar standing long-leg radiographs are typically performed for the measurement of dimensions, distances and angles with the advantage of imaging the lower limbs in their naturally loaded, physiologically relevant position.

However, traditional cone-beam imaging technologies demonstrate limitations [1], most importantly image distortion, in particular towards the long ends of the plate [2].

With the advent of digital radiography (DR) and its smaller detector sizes, image stitching has increasingly been used, reducing the amount of projectional distortion but in turn introducing the possibility of stitching artifacts [3]. Because the image is bifocal or trifocal, any correction or accounting for such distortional effects is impaired.

Furthermore, CR and DR techniques do not consider the spatial depth of a three-dimensional structure. Three-dimensional standard computed tomography would be capable of overcoming these problems, but critically cannot provide imaging in a naturally loaded, physiological alignment.

The more recently developed EOS 2D/3D Imaging system (EOS imaging, Paris, France) was designed to acquire synchronous, bi-planar long-leg standing images. Based on its slot-scanning technology, the EOS system is distortion-free and true to dimension in the vertical direction [4]. The synchronous and orthogonal image acquisition protocol of EOS permits for an automatic, reciprocal distortion correction in the antero-posterior and medio-lateral directions [5].

The aim of this descriptive study was to investigate the measurement differences between CR, DR and EOS when acquiring long-leg standing images in the coronal plane in several clinically important measures.

## Materials and methods

### Study design

This is a prospective, non-interventional, multi-arm, descriptive radiological study on a human hemiskeleton.

### Human hemiskeleton, instrumentation, positioning

**Fig. 1:**
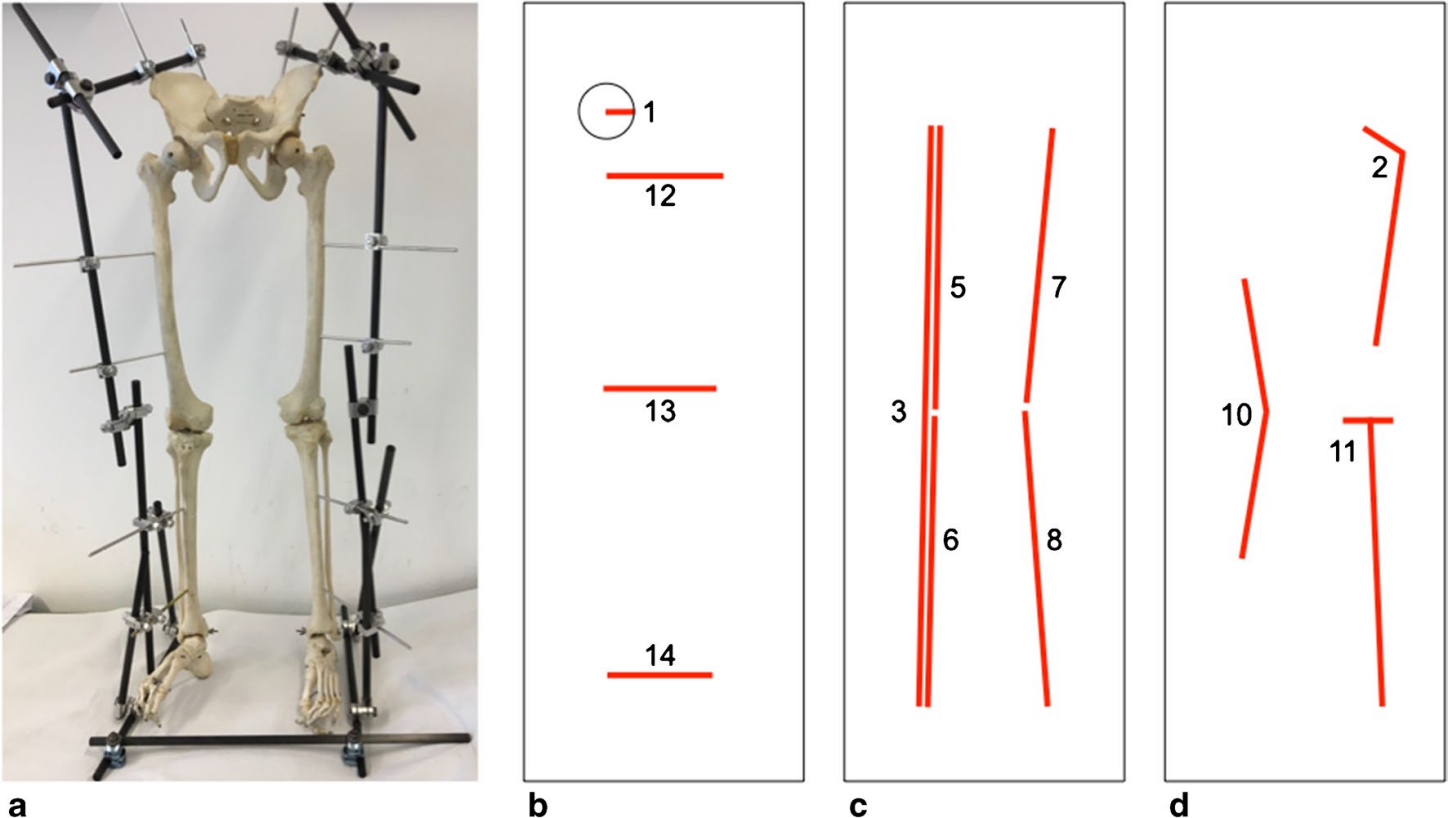
Instrumented hemiskeleton measurements. **a** The assembled hemiskeleton with the external fixator in neutral alignment. **b** The horizontal distance measures as well as the measurement principle for the femoral head radius. **c** The vertical measures. **d** The angle measures

A partial human skeleton (pelvis and both complete lower extremities was instrumented by an experienced Orthopedic Surgeon (CB) using a trauma external fixator consisting of Schanz pins in surgical steel and carbon bars (DePuy Synthes, Umkirch, Germany) such that the spatial relationships between pelvis, femora and tibiae could be freely adjusted and then rigidly fixed (Fig. 1a). The system was constructed in a way as to incorporate 3 bars at the base that would permit the instrumented skeleton to stand freely and in a stable, reproducible position (Fig. 1a).

In order to minimize any projectional phenomena resulting from the configuration of the skeleton itself, care was taken to align the femora and the tibiae in a single coronal plane as closely as possible for the neutral, the varus, and the valgus alignments. For varus alignment, the knee joints were symmetrically spread apart as wide as the narrowest image detector could accommodate, which resulted in a varus angle ranging from 15° to 20° when measuring with a goniometer in a pure frontal plane. For valgus alignment, the knee joints were symmetrically approximated towards each other to an extent, where in a flesh-covered body the soft tissues would be expected to touch, resulting in a valgus angle of approximately 15° when measuring with a goniometer in a pure frontal plane.

### Imaging

The assembled skeleton was imaged in a standing position with neutral, varus and valgus knee alignments. Four different imaging systems were utilized:

- a traditional CR system (Siemens AXIOM Multix M TOP) with a single long plate of 83.7-cm length in the long dimension and with a distance of 300 cm from the radiation source to the film. In the manuscript text, this modality is termed “CR.”
- a typical DR system (Siemens YSIO MAX Aim Fast) with a digital detector of 43-cm length in the long dimension and with a distance of 300 cm from the radiation source to the film. In the manuscript text, this modality is termed “DR.”
- a more recent DR variety (Siemens Ysio Model 10281013) with a distance of 300 cm from the radiation source to the film. In the manuscript text, this modality is termed “Ysio.”
- an EOS® 2D/3D system (EOS Imaging, Paris, France) with 2 x-ray tubes and 2 Charpak gaseous detectors in orthogonal alignment. The distance from the radiation source to the detector in this system is 130 cm with the subject positioned in the center. In the manuscript text, this modality is termed “EOS.”

To control for the typical variables that are immanent to routine clinical imaging such as precise subject positioning, X-ray beam adjustment, central beam alignment, etc., 10 images were acquired for each anatomical alignment (3 variations) and for each imaging system (4 systems), resulting in a total of 120 images. For each exposure, the skeleton was removed and repositioned by means of visual control in reference to the detector plane and the imaging system used was newly adjusted from its zero position. As this study aimed to demonstrate potential differences between several imaging techniques that impact everyday clinical practice, we decided to perform the actual imaging as close to clinical routine as possible, positioning the patient with the help of and according to the visual assessment of a trained radiography technician for each imaging device. Doing so not only insured comparing the 4 different imaging systems in to each other, but rather investigating them in their clinically relevant operational mode. Once all 40 images for a given anatomical position had been acquired, the external fixator was loosened and the next anatomical position was set before the hemiskeleton was imaged with all 4 imaging systems.

### Image acquisition and processing

Each image that was acquired in the context of this study was labeled with an individual and unique research ID. The image data from the 4 different imaging devices was saved to the hospital PACS system in standard DICOM format. For data analysis, all images were queried and loaded into a sterEOS® workstation (EOS Imaging, Paris, France) that was attached to a certified radiology analysis monitor. The measurements were performed on sterEOS software, but only using manual 2D measurement tools. No 3D tools were used in this experiment. All measurements were manually entered into a specifically designed MS Excel database (Microsoft, Munich, Germany).

### Radiological measurements

All images were manually measured according to a predesigned analysis workflow that allowed for an efficient, non-repetitive and error-avoiding placement of the pertinent anatomical markers while zooming in on the anatomical structures of importance. Measures were performed using a line tool for distances, 2 lines and an automated angle calculation tool for angles and a dedicated circle tool (based on 3 reference points and automatically generating the center point) for the femoral head radius. The circle tool used in this study relies on 3 anatomic markers and therefore permits for the very precise creation of a circle that best matches the circumference of the femoral head as it is projected in each individual image. All measurements were performed according to established and published radiological standards by a single examiner with more than 20 years of experience [6]. Because image distortion and projection artifacts might affect different regions in an image, and affect these regions differently according to the imaging technology used for acquisition, a range of clinical measures were studied for each side (left and right) (see details in Fig. 1b–d):

1. femoral head radius (mm)
2. CCD angle (°)
3. distance from the center of the femoral head to the center of the tibiotalar joint of the same side (mm)
4. functional leg length calculated as the sum of the above measure and femoral head radius (4 = 3 + 1, mm)
5. functional femoral length (mm, derived from the Mikulicz line measuring from the center of the femoral head to the point where this line intersects with the mid-articular plane of the knee joint)
6. functional tibial length (mm, derived from the Mikulicz line measuring from where this line intersects with the mid-articular plane of the knee joint to the end point at the tibial plafond)
7. anatomical femoral length (mm, measured from the center of the femoral head to the anatomical center of the knee joint)
8. anatomical tibial length (mm, measured from the anatomical center of the knee joint to the mid-point of the tibial plafond)
9. anatomical leg length calculated as the sum of the anatomical femoral and tibial lengths (mm)
10. varus or valgus angulation between the anatomical axes of femur and tibia (°, with negative values indicating a valgus angle and positive values indicating a varus angle)
11. anatomical medial proximal tibial angle (MPTA, °)
12. horizontal distance between both lesser trochanters (mm)
13. horizontal distance between both tibial condyles (mm)
14. horizontal distance between both medial malleoli (mm)

In total, 1680 measures were acquired from 120 radiological images. In addition to anatomical measures of routine clinical importance, 3 distance measurements in the horizontal plane (measurements 12 – 14) were added in order to permit a comparison of the projectional distortion in the horizontal plane between the 4 imaging modalities. The upper and lower periphery of the image was evaluated by measures 1, 2, 12, and 14, while the center was evaluated by measures 10, 11, and 13. Measures 3, 4, and 9 were global while measures 5 to 8 concentrated only on one half of the image. Measurement 10 was exceptional as it is an angle measurement based on two long bones, one from each half of the image (femur – upper, tibia – lower).

### Supplemental mechanical measurements

The diameters of the two femoral heads were measured with a precision caliper as a reference. Based on their spherical shape, they were the only anatomical dimension of the skeleton that could be measured entirely without the influence of positioning / projection onto a single plane and without requiring at least partial destruction of the hemiskeleton.

Both femoral and tibial anatomical lengths were measured mechanically using an orthopedic tape measure placed on the anterior cortex of the femur from the upper cortex of the femoral head to the center of the trochlea (and while deducting the femoral head radius from the actual measurement) and from the intercondylar eminence to the center of the tibial plafond, respectively. Femoral antecurvation and the non-linearity of the anterior tibial cortex, however, render these latter measurements slightly less reliable in comparison to the femoral head measurements. Values for femoral head diameter, anatomical femoral and tibial leg lengths were inserted into the respective figures as a reference line for validation of imaging methods.

### Data analysis and visualization

In order to visualize our data in an easily accessible fashion, these are presented in the form of “box-and-whisker” charts. The only exception to this mode of data presentation is for the measurements of the femoral head radius where a column graph for mean values with error bars representing the standard deviation of each individual data set was chosen. Box-and-whisker charts: The upper and lower limits of each box represent the upper and lower quartiles of data distribution (25th and 75th percentile, respectively) whereas the whiskers indicate the maximum and the minimum values. A horizontal line within the box represents the median and any outliers are shown as individual dots above or below the ends of the whiskers.

When differences between groups with different imaging modalities or with different anatomical alignments were tested for statistical significance, the two-tailed *t*-Test for unrelated samples was used and a *p*-value of < 0.05 was defined as statistically significant (alpha 0.05).

## Results

### Distance measurements

#### Femoral head radius

**Fig. 2:**
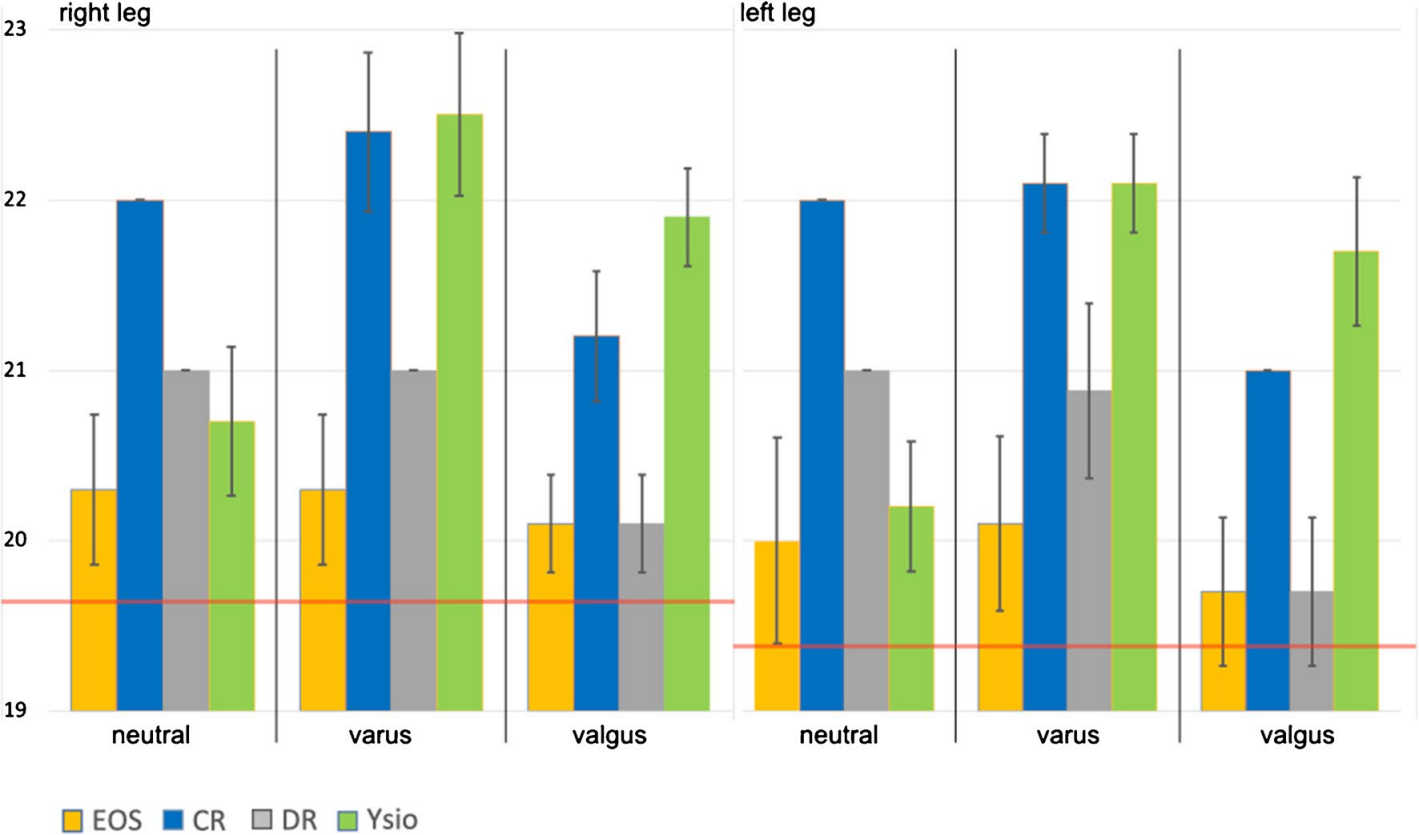
Femoral head radius (mm). Column graph displaying the means for the left and the right femoral head radius, for neutral, varus and valgus alignments as well as with the 4 different modalities. Each column represents a mean value, whereas the error bar stands for the standard deviation of the individual data series. The horizontal red lines represent the mechanical measurements for each femoral head

When comparing modalities, EOS measurements of the femoral head radii consistently were the smallest and most accurate compared to the mechanical measurements (Fig. 2), whereas CR measurements were the largest. When comparing the measurements between neutral, varus and valgus alignments within a single modality and for each side separately, only EOS measurements were not significantly different. This means, that only EOS yielded femoral head radius measurements that were without exception independent of the anatomical alignment. EOS measurements were the most consistent, smallest and closest to the true anatomical dimensions as objectively determined with a caliper.

#### Functional leg length

**Fig. 3:**
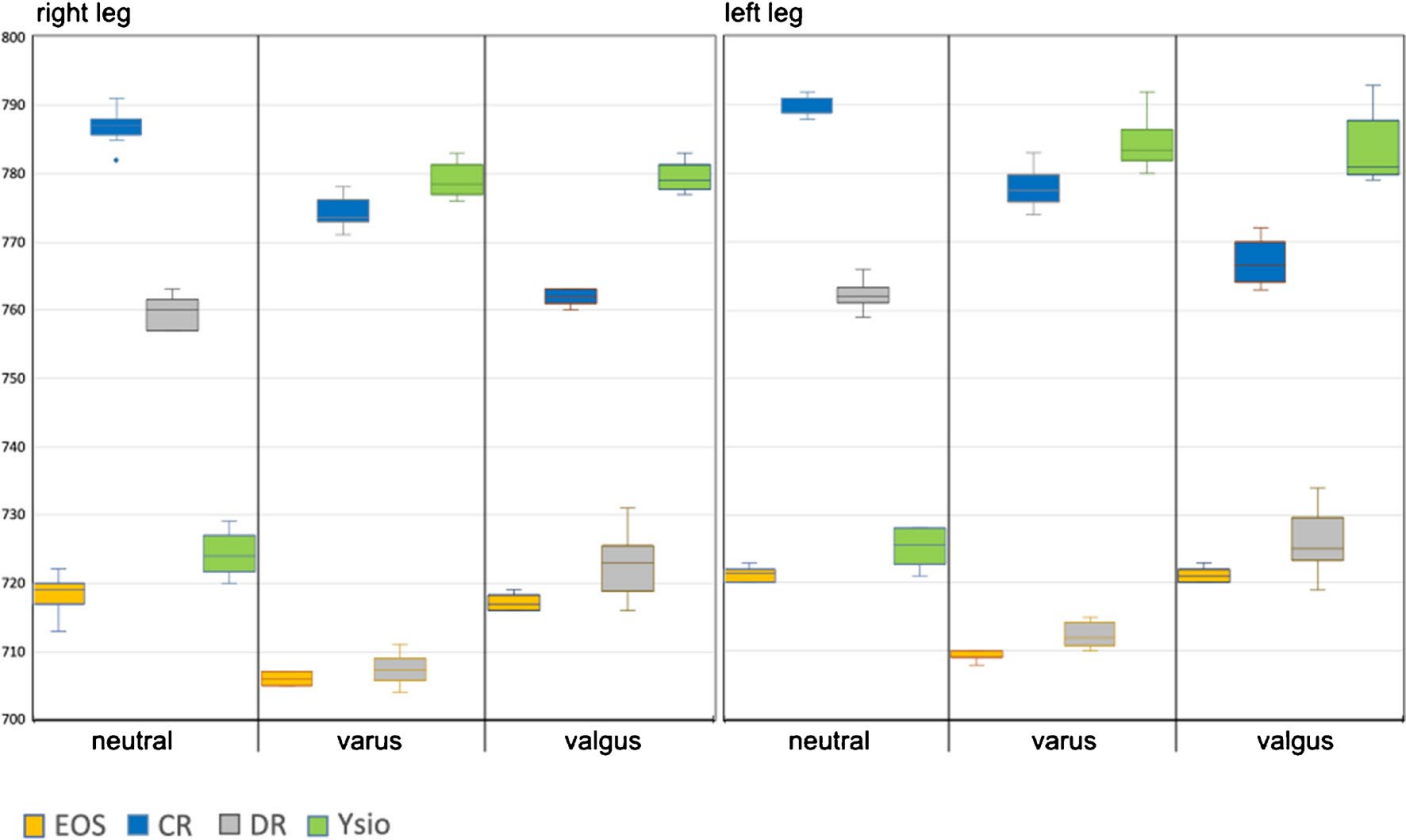
Box-and-whisker graph for the functional leg length (mm), as calculated from femoral head radius + functional femoral length + functional tibial length

The differences observed between modalities were comparatively large, most likely due to the large distance measured. However, EOS measurements consistently resulted in the shortest and most accurate distances compared to mechanical measurements (Fig. 3). The remaining modalities demonstrated statistical differences, both intramodal within different alignments as well as intermodal.

#### Anatomical tibial and femoral length

**Fig. 4:**
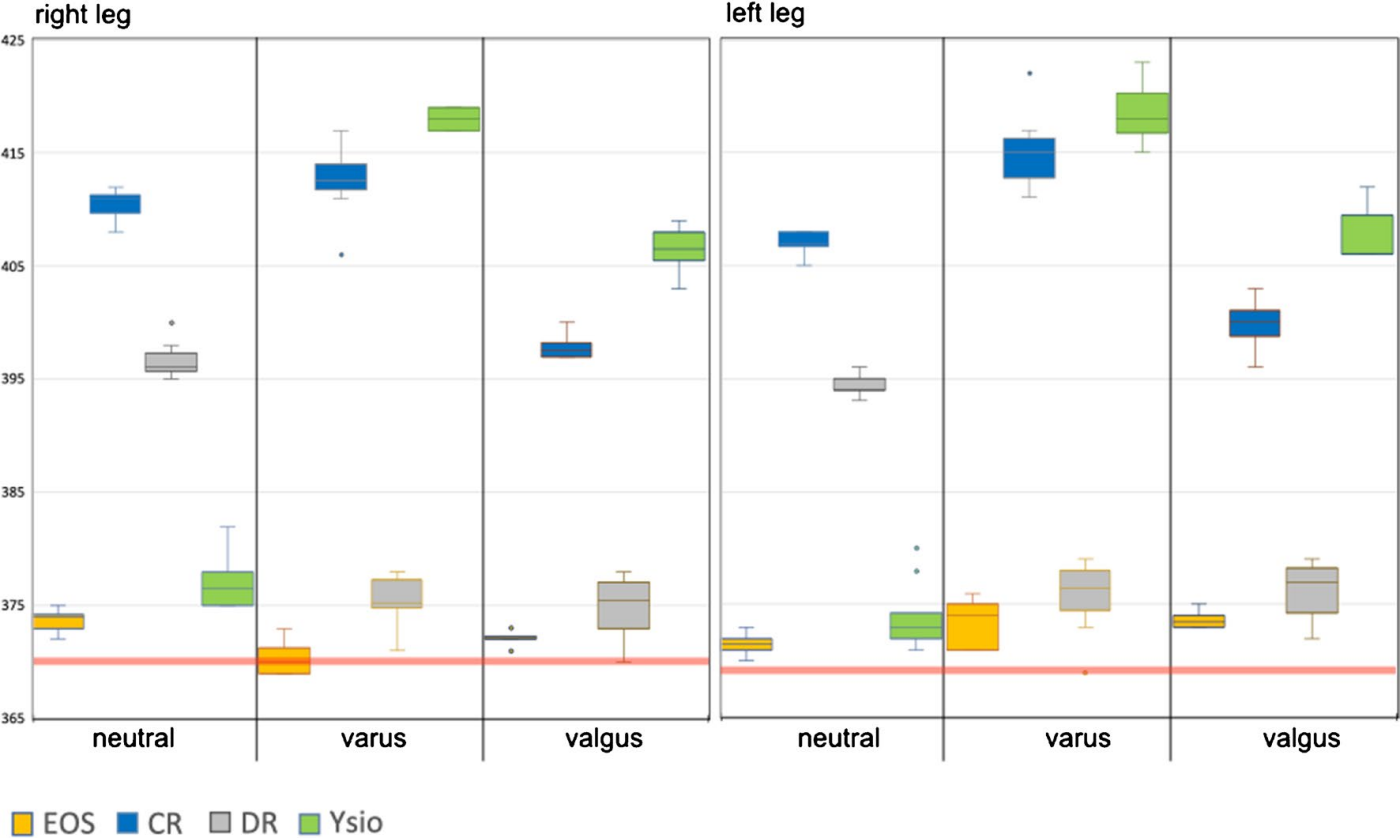
Box-and-whisker graph for anatomical femur length (mm). The horizontal red lines represent the mechanical measurements for each femur

**Fig. 5:**
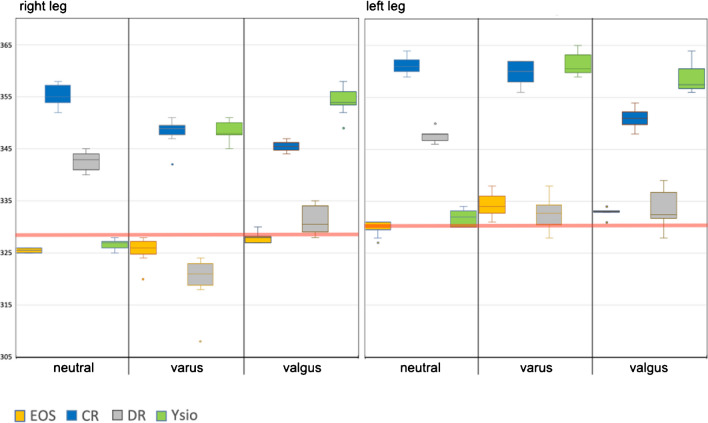
Box-and-whisker graph for anatomical tibia length (mm). The horizontal red lines represent the mechanical measurements for each tibia

Observations for the different imaging modalities (Figs. 4 and 5) were in principle identical to the ones explained in the preceding paragraph since their sum correlates to functional leg length. As Fig. 4 displays, a longitudinal distance measure (anatomical femoral length) that theoretically should yield identical measurements independent of varus or valgus alignment and also independent of the modality used, resulted in measurement differences as large as 54 mm, which equates to a 13.6% (of the mean) difference between minimum and maximum measurements. The differences between the individual modalities within a given anatomical alignment group were all statistically highly significant with p-values much smaller than 0.001. We then also examined the differences within a given imaging modality, but between anatomical alignments. Only EOS resulted in almost identical measurements for all 3 alignments, and these measurements also correlated closest with our direct mechanical measurement as depicted in Figs. 4 and 5.

#### Distance between the lesser trochanters, tibial condyles, and medial malleoli

**Fig. 6:**
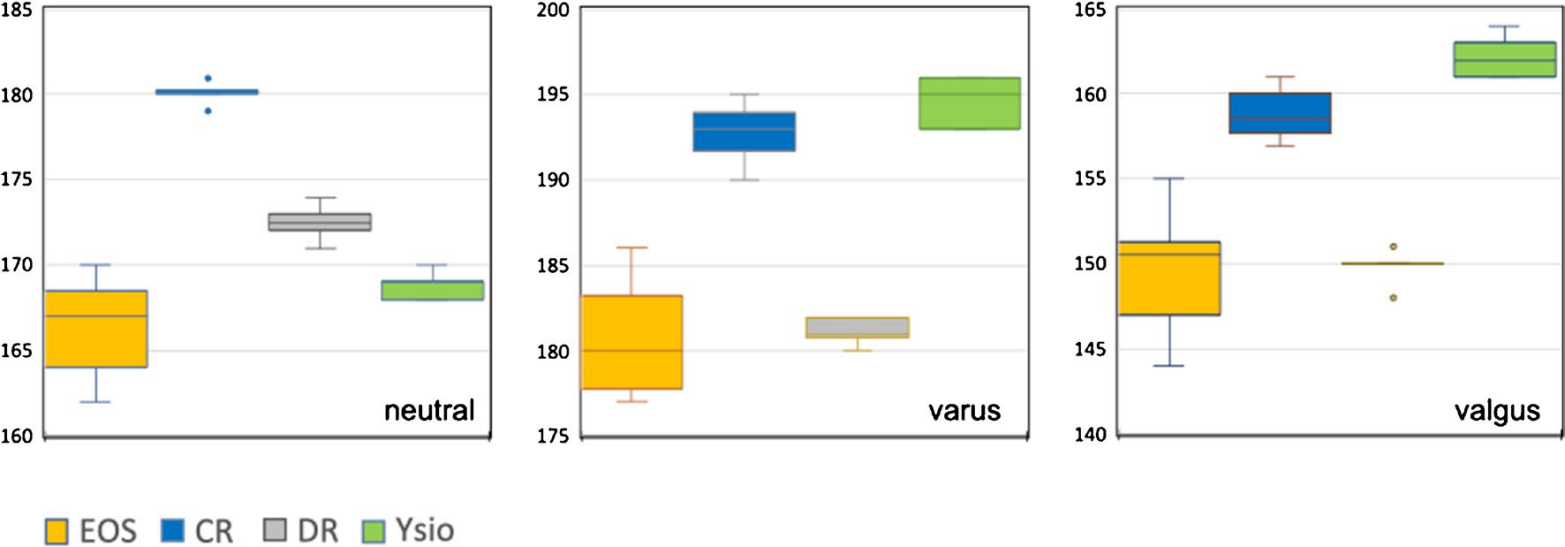
Box-and-whisker graph for the distance between the lesser trochanters (mm)

**Fig. 7:**
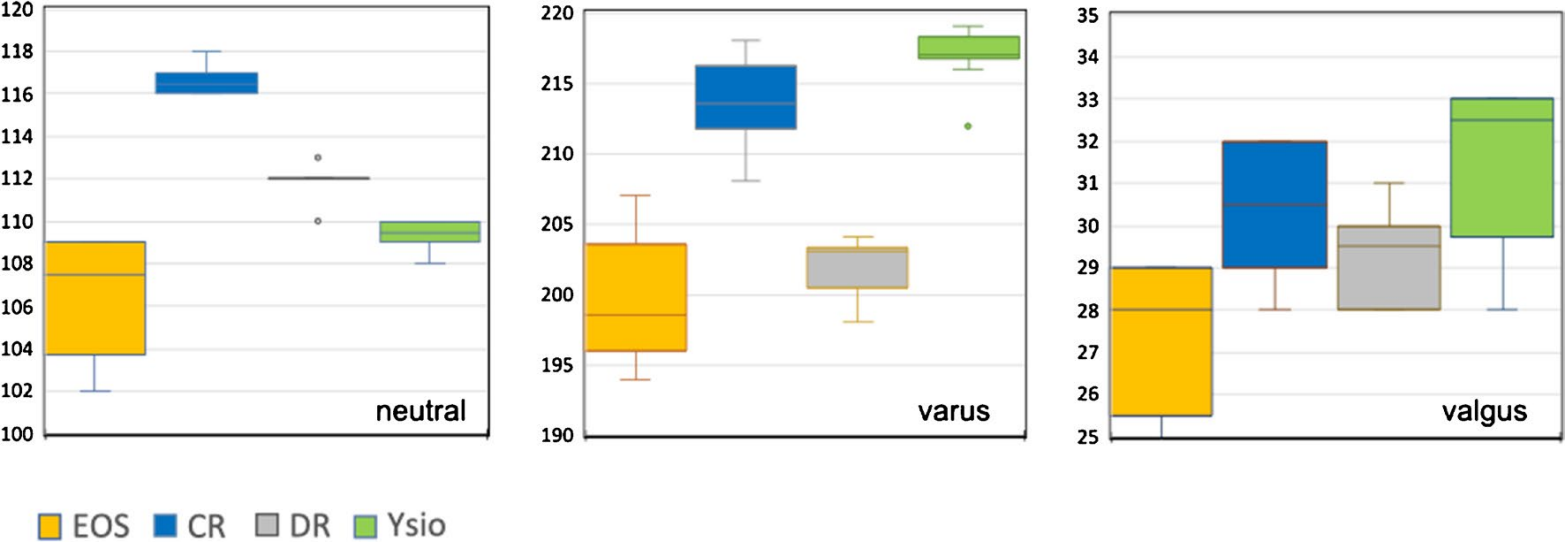
Box-and-whisker graph for the distance between the tibial condyles (mm)

**Fig. 8:**
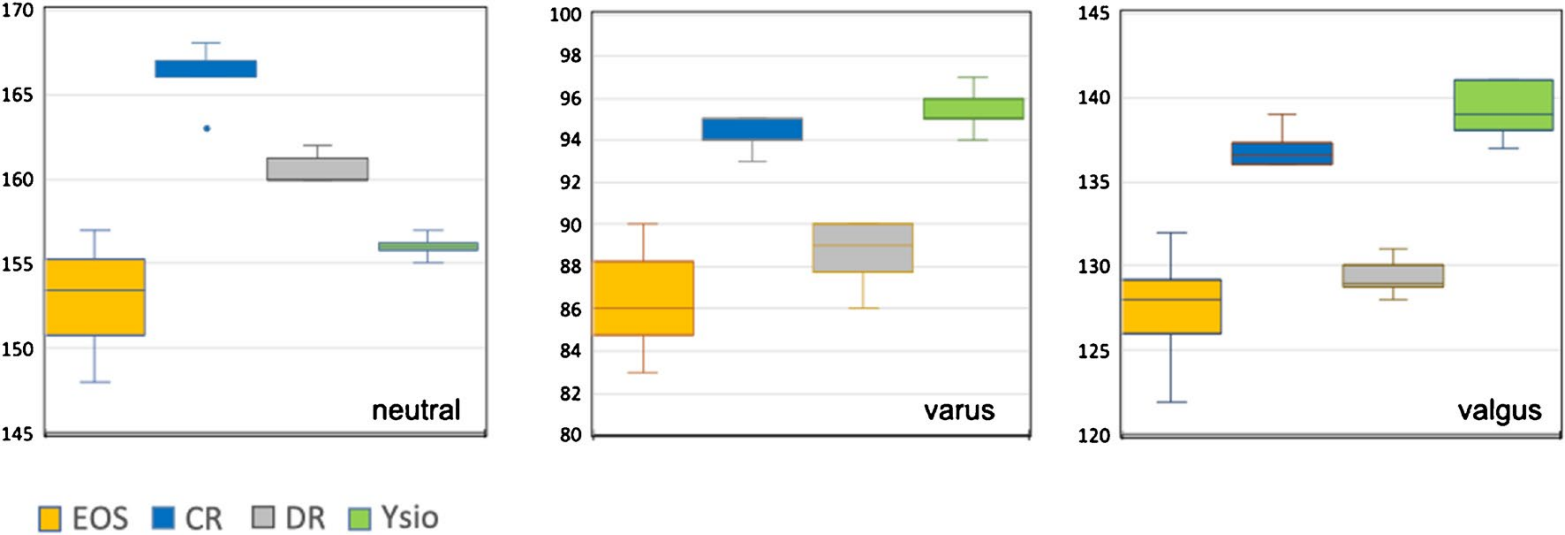
Box-and-whisker graph as for the distance between the medial malleoli (mm)

Also with these horizontal distances, EOS measurements consistently were the shortest (see Figs. 6, 7, and 8). The differences between modalities for neutral alignment were all statistically significant.

### Angle measurements

#### CCD angle

**Fig. 9:**
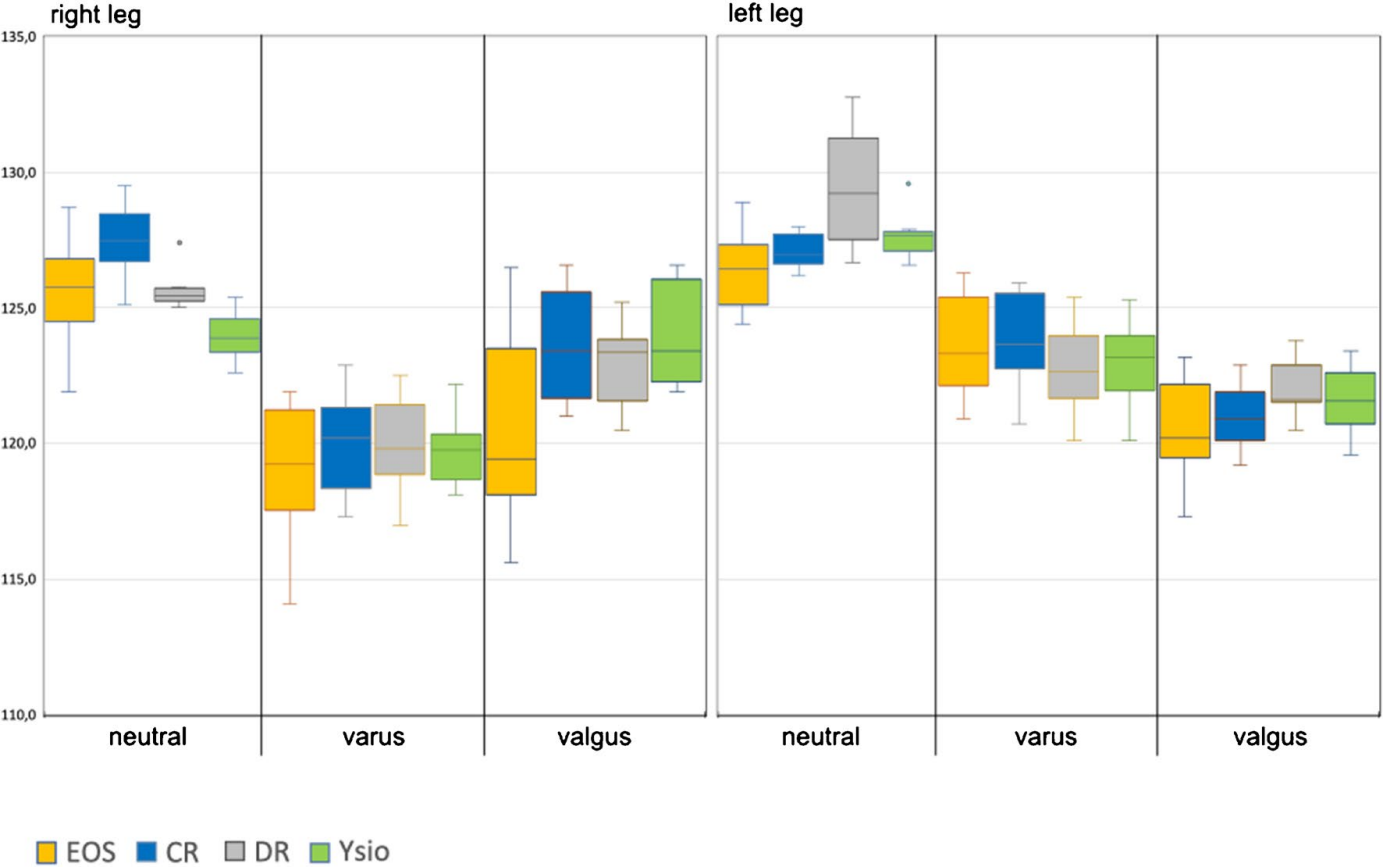
Box-and-whisker graph for the CCD angle (°)

We found large differences (Fig. 9) with a 14° spread of measurements on an anatomical measure that had an absolute mean of 125.8° for the left and of 124° for the right side, equating to a measurement imprecision of 11.1% and of 11.2% between these 4 modalities. Furthermore, the variance within the individual groups was also rather large in comparison to some of the distance measurements with most significant differences in neutral alignment. However, in varus alignment, none of the differences were statistically significant and in valgus alignment, only individual comparisons between modalities demonstrated significant differences.

#### Varus – valgus alignment of femur and tibia

**Fig. 10:**
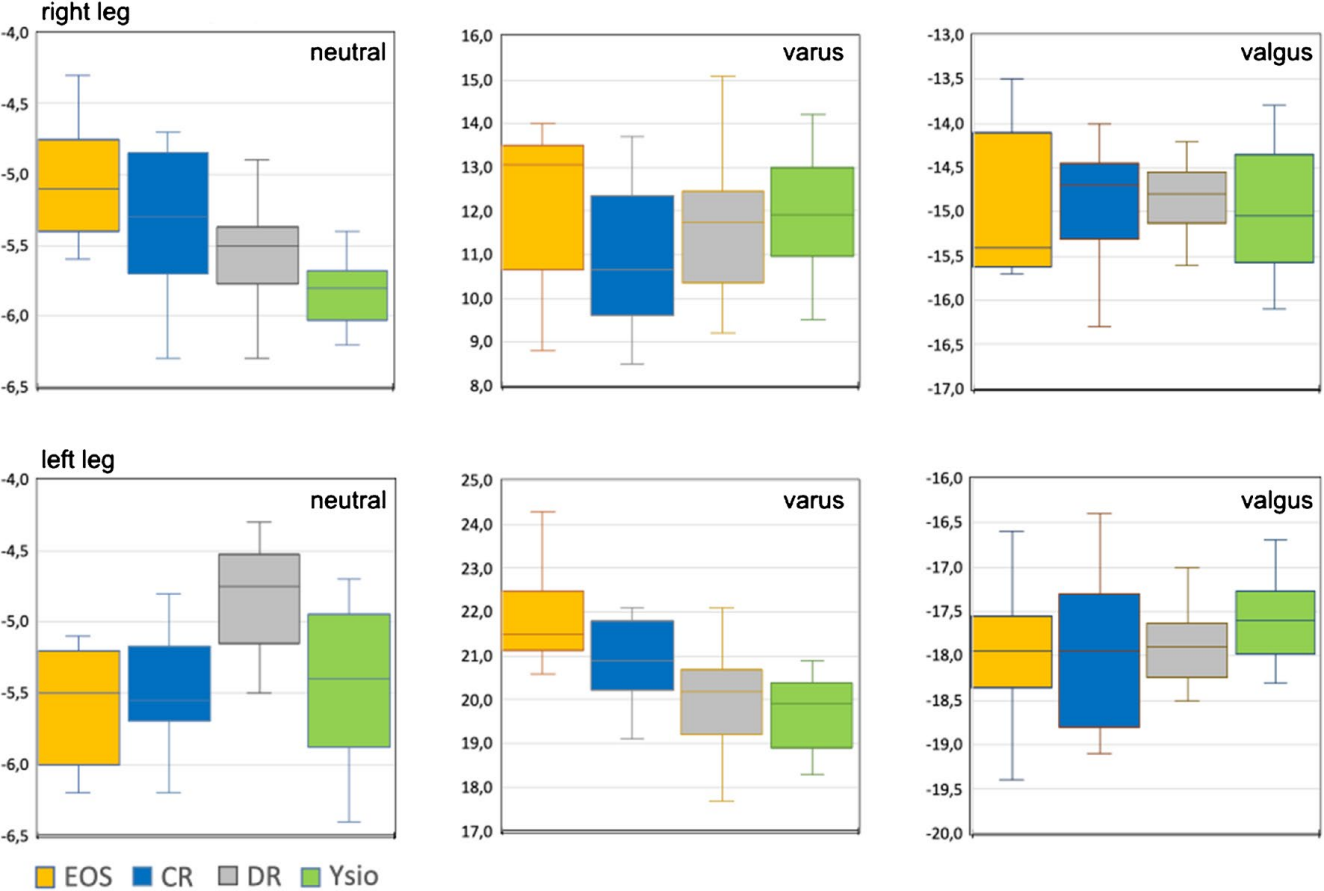
Box-and-whisker graph for the varus or valgus alignment of the knee joints (°)

With neutral and with valgus alignment the data ranges of the 4 modalities largely overlapped (Fig. 10) with an intermodal range between 2° and 3°. With varus alignment, the intermodal spread was more than 7° also with intermodal largely overlapping data ranges. The differences between modalities and within alignment groups and sides were mostly statistically non-significant with individual deviations in neutral and varus alignments.

#### MPTA

**Fig. 11:**
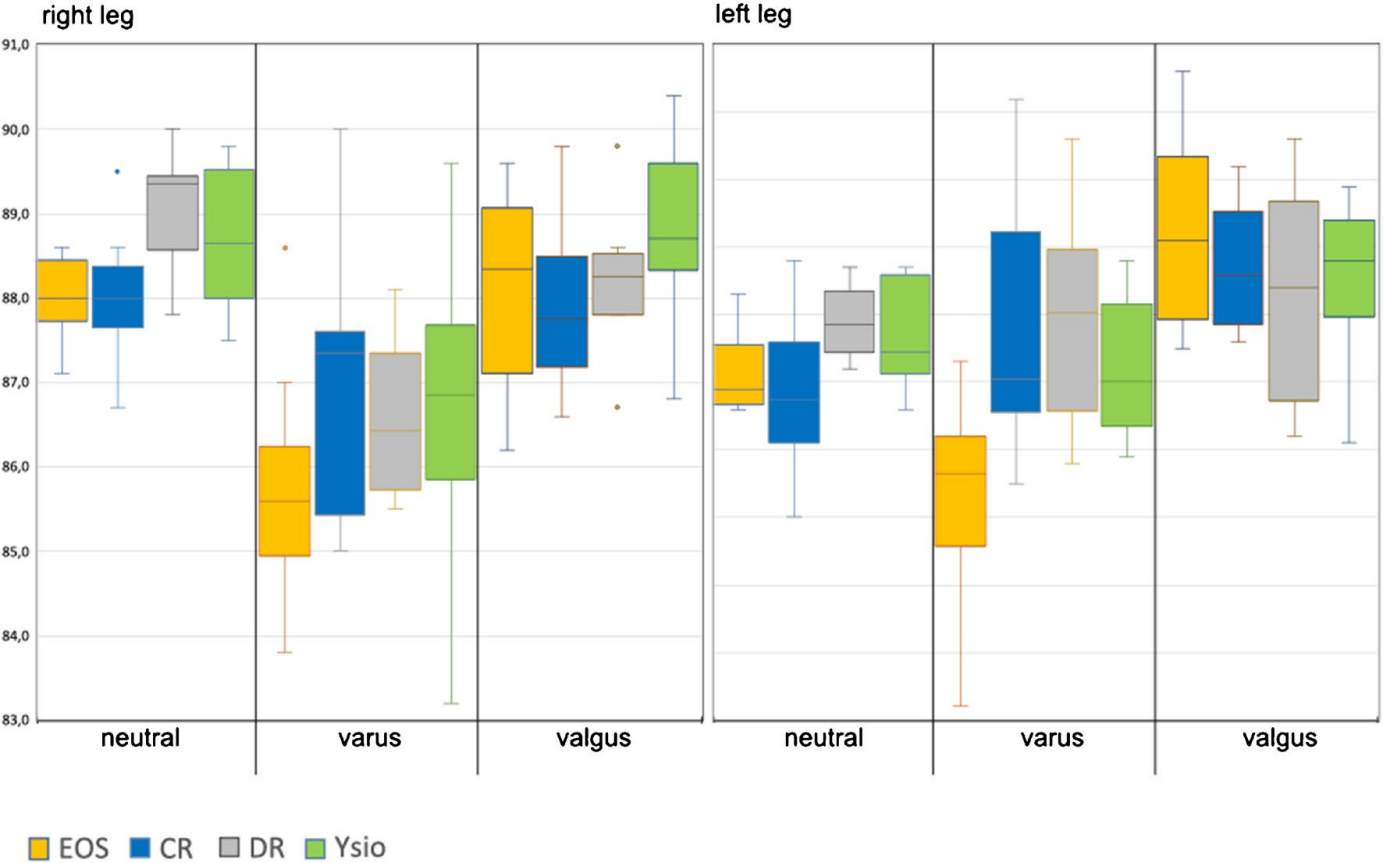
Box-and-whisker graph for the anatomical medial proximal tibial angle (MPTA, °)

Our measurements consistently fell within the published normal range of between 85° and 90° [6], with 87° being the median and we did not observe statistically significant differences between the modalities (Fig. 11).

## Discussion

The EOS system employs two very narrowly collimated fan beams and detector systems, orthogonally aligned towards each other in the horizontal plane and moving synchronously from top to bottom during image acquisition. Based on these characteristics, the vertical plane is imaged true to size by means of the physical process alone [7]. The remaining fan beam-related distortion in the horizontal plane is corrected by the internal image processing algorithm based on the positional information gained from the lateral plane image. Our data showed that EOS measurements were closest to the true anatomical dimensions as measured by means of a caliper for the femoral head diameter as well as anatomical measurements of both femur and tibia as well as functional leg length.

Precise measurements of distances and angles in standing long-leg radiographs are essential as slight differences can have a substantial impact on treatment outcome, e.g., hip or knee replacement, which are the most commonly performed orthopedic procedures in the USA with steep projected increases in numbers over the next decades [8].

Concerning vertical distance measurements, only EOS consistently delivered results that showed no statistically significant differences between neutral, varus and valgus alignments, demonstrating a higher technical precision over the other three modalities towards the upper end of a long-leg-image. The influence of projectional distortion in the vertical direction was particularly obvious with functional leg length where EOS consistently delivered the shortest measurements as opposed to CR with the longest measurements. EOS measurements also were by far the most consistent between the 3 anatomical alignments with less than 15-mm difference between the extremes (Fig. 3).

The horizontal distance measurements showed the same discordant results in between modalities.

The differences in femoral head radius measurements between EOS and CR are very likely explained by the larger projectional distortion with CR towards the upper end of the film as opposed to the EOS measurements being physically precise in the vertical direction. Our data therefore confirm the accepted clinical practice, that precise component planning for total hip arthroplasty cannot reliably be performed on long-leg films, except on EOS images, without a calibration device in the immediate neighborhood and in the same plane as the femoral head itself [9, 10]. And if long-leg films are used for such or similar applications, based on physical-technical grounds, our data as well as other research suggest that EOS provides less variability between patients’ positions, indicating that it provides more coherent values than the other modalities [10].

Correspondingly, we observed the largest effect of projectional distortion for the intermalleolar distance, which is the horizontal measure that is located farthest from the central beam of a cone beam. The physical-optical situation at the upper or lower end of an EOS image are identical to the situation at the center of an image, which is not impacted (for vertical) or corrected (for horizontal) from projectional distortion and which is a fundamental difference to all cone beam techniques, which might impact surgical planning of distal lover limb osteotomies or analyses of foot and ankle statics.

With regards to angular measurements, we observed only minor, but statistically significant differences between the modalities. Angular measurements are typically subject to stronger projectional distortion than distance measurements [11].

The CCD angle is of considerable clinical importance in Orthopedic Surgery and the limitations of a two-dimensional measurement of this angle that anatomically lies not within the coronal plane has been discussed previously in the literature [12, 13].

Means of measurements within each anatomical alignment group showed no statistically significant difference between modalities. That given and based on our data, CCD measurements in uniplanar radiography appear to be too unreliable for precise surgical planning and this aspect deserves further investigation. Angular distortion with projectional radiography has been demonstrated to be a problem in certain applications and our research adds to this concern [14].

The measurements for varus and valgus alignment of the knee showed only very small intermodal differences in neutral and in valgus alignment. However, spread of individual measurements within each modality for an identical anatomical situation were rather large and beyond the degree of precision required for clinical decision making (1° between means and less than 3° for the right knee in varus alignment), which would be a strong argument for a 3-dimensional analysis.

With regards to MPTA, measurement ranges exceeded the reported normal ranges, particularly at the lower end, placing the clinical reliability of such measurements for the planning of corrective tibial osteotomies alone into question.

Our results align with previous studies concerning long-leg radiographs and the EOS system: Earlier studies showed that the EOS system can depict specific 3-dimensional characteristics of lower extremities and its results correlate with CT examinations [15]. This is important as even in healthy subjects, lower extremity alignment can vary significantly [16]. When compared to CR, EOS has shown differences in varus/valgus angle, femoral length as well as total limb length [17]. This matches our results for functional leg length as well as anatomical tibial and femoral length with significant intermodal differences. Varus alignment also demonstrated the greatest variance in our study, but without statistical significant differences between modalities. However, another study on patients with knee osteoarthritis found no significant difference in measurements between CR and EOS [18]. After total knee replacement, some studies have also indicated reliable measurements for EOS when compared to CT [19], but again with considerable variances for varus/valgus alignment [20].

As one limitation to our study and except for femoral head radius, we cannot provide “absolute true” horizontal distances or angular measurements. Even mechanical measurements of the anatomical tibial and femoral length as well as functional leg length were obtained with a certain degree of incorrectness because of anatomical characteristics like the curvature of the femur, respectively.

In summary and when comparing the performances of these 4 imaging modalities for uniplanar measurements, the EOS imaging system delivered by far the most consistent and physically correct results for distance measurements in terms of the independence from different anatomical alignments and from the region of a long-leg-image.

However, the EOS system in simple 2D-mode (meaning: without the use of the EOS system’s proprietary 3D-modeling and -measuring) can also not overcome the shortcomings that result from 3-dimensional structures being measured in a 2-dimensional projection plane based on our results as well as shown in previous studies. Nevertheless, we need to accept, that uniplanar measurements represent the current clinical standard, which we had set out to examine with this study.

It should be highly interesting for future research to compare a 3-dimensional analysis of such EOS images with measurements taken within the 3-dimensional space of computed tomography scans as was done in other areas previously [21–23].
